# Stability of Gut Enterotypes in Korean Monozygotic Twins and Their Association with Biomarkers and Diet

**DOI:** 10.1038/srep07348

**Published:** 2014-12-08

**Authors:** Mi Young Lim, Mina Rho, Yun-Mi Song, Kayoung Lee, Joohon Sung, GwangPyo Ko

**Affiliations:** 1 Department of Environmental Health, Graduate School of Public Health, Seoul National University, Gwanak-gu, Seoul, Republic of Korea; 2 Division of Computer Science and Engineering, College of Engineering, Hanyang University, Seongdong-gu, Seoul, Republic of Korea; 3 Department of Family Medicine, Samsung Medical Center, Sungkyunkwan University School of Medicine, Gangnam-Gu, Seoul, Republic of Korea; 4 Department of Family Medicine, Busan Paik Hospital, Inje University College of Medicine, Busan Jin-Gu, Busan, Republic of Korea; 5 Department of Epidemiology, Graduate School of Public Health, Seoul National University, Gwanak-gu, Seoul, Republic of Korea; 6 Center for Human and Environmental Microbiome, Seoul National University, Gwanak-gu, Seoul, Republic of Korea; 7 N-Bio, Seoul National University, Gwanak-gu, Seoul, Republic of Korea

## Abstract

Studies on the human gut microbiota have suggested that human individuals could be categorized into enterotypes based on the compositions of their gut microbial communities. Here, we report that the gut microbiota of healthy Koreans are clustered into two enterotypes, dominated by either *Bacteroides* (enterotype 1) or *Prevotella* (enterotype 2). More than 72% of the paired fecal samples from monozygotic twin pairs were assigned to the same enterotype. Our longitudinal analysis of these twins indicated that more than 80% of the individuals belonged to the same enterotype after about a 2-year interval. Microbial functions based on KEGG pathways were also divided into two clusters. For enterotype 2, 100% of the samples belonged to the same functional cluster, while for enterotype 1, approximately half of the samples belonged to each functional cluster. Enterotype 2 was significantly associated with long-term dietary habits that were high in dietary fiber, various vitamins, and minerals. Among anthropometrical and biochemical traits, the level of serum uric acid was associated with enterotype. These results suggest that host genetics as well as host properties such as long-term dietary patterns and a particular clinical biomarker could be important contributors to the enterotype of an individual.

Trillions of microbes reside in the human gut, carrying out functions that are critical to host health, including dietary energy extraction, development and maintenance of the immune system, and protection against pathogens^1^,^2^,^3^. The composition of the gut microbiota is known to vary greatly among healthy individuals^4^,^5^, and to be influenced by various factors such as age, nutrition, and geography^6^,^7^. In addition, compositional and functional changes in the gut microbiota have been observed in patients with several chronic disorders including obesity, type 2 diabetes, and inflammatory bowel disease^2^,^8^,^9^.

Arumugam *et al.* suggested that human gut microbial communities can be clustered into three enterotypes, each of which is represented by different dominant genera: *Bacteroides*, *Prevotella*, or *Ruminococcus*. These enterotypes were not significantly correlated with host properties such as nationality, age, gender, or body mass index (BMI)^10^. Subsequent studies have demonstrated associations of enterotypes with long-term dietary patterns^11^ and several diseases such as symptomatic atherosclerosis^12^ and nonalcoholic steatohepatitis^13^. Recently, it has been somewhat controversial about the number and discreteness of enterotypes^11^,^14^,^15^,^16^. A recent study using Human Microbiome Project (HMP) data showed that the gut microbiota is represented as a bimodal distribution and smooth gradient rather than a discrete distribution^16^. More recently, Arumugam *et al.* stated in the addendum of their original enterotype paper that enterotypes should be considered as a way to simplify the complexity of the gut microbiota rather than as distinct clusters^17^. So far, however, it is unknown whether the enterotypes that were studied mainly in populations from Europe and the United States^10^,^11^,^18^ are present in an Asian population with different dietary and genetic backgrounds. In addition, whether enterotypes are influenced by host genotype, or how stable enterotypes are over time, remains uncertain.

Here, we conducted taxonomic and functional profiling of gut microbiomes from healthy Korean monozygotic (MZ) twins to characterize their enterotypes and functional clusters, and to determine the presence or absence of genetic effects on enterotypes as well as the longitudinal stability of enterotypes in a Korean population. In addition, we further investigated associations of enterotypes with long-term dietary habits and clinical biomarkers.

## Results and Discussion

### Clustering Korean gut microbiome into enterotypes

To investigate whether the Korean population could be grouped into enterotypes, we applied the partitioning around medoids (PAM) method using Jensen–Shannon (JS) distance for the genus-level relative abundance profiles from metagenomic shotgun sequencing data, following the procedure of the previous study^10^. Recently, Koren *et al*. recommended using at least two distance metrics because of the sensitivity of enterotyping to the distance metrics^16^. Therefore, we also applied the two other distance metrics (Bray–Curtis [BC] and Euclidean [EU]). To determine the optimal number of clusters and evaluate the cluster quality, we calculated the Calinski–Harabasz pseudo F-statistic (CH) value and the Rousseeuw's Silhouette internal cluster quality index (SI) for each of three distance metrics as a relative and an absolute measure, respectively, recommended by Koren *et al*.^16^. The highest CH value was obtained for two clusters for all of the tested distance metrics. For two clusters, SI provided weak support (0.25 < SI ≤ 0.5) using EU distance, and no support (SI ≤ 0.25) using JS and BC distances according to the criteria proposed by Kaufman and Rousseeuw^19^ (Supplementary Fig. S1). Interestingly, we observed that the sample assignments to clusters obtained by using JS and EU distances were perfectly identical (Supplementary Table S1). We thus inferred that these two distance metrics formed reliable clusters from our metagenome data, and chose to use the two clusters generated using JS distance for further analyses, as in Arumugam *et al*.^10^. A study using HMP data showed that the sample size could affect the number of enterotypes^20^. In this regard, our results should be interpreted with caution due to the relatively small sample size. However, our results were consistent with a number of previous studies with larger sample sizes. In these studies, two enterotypes were also observed^11^,^14^, and a recent study by Koren *et al*. indicated the bimodal distribution of gut microbiota^16^.

### Compositional and functional characteristics of enterotypes

**Figure 1:**
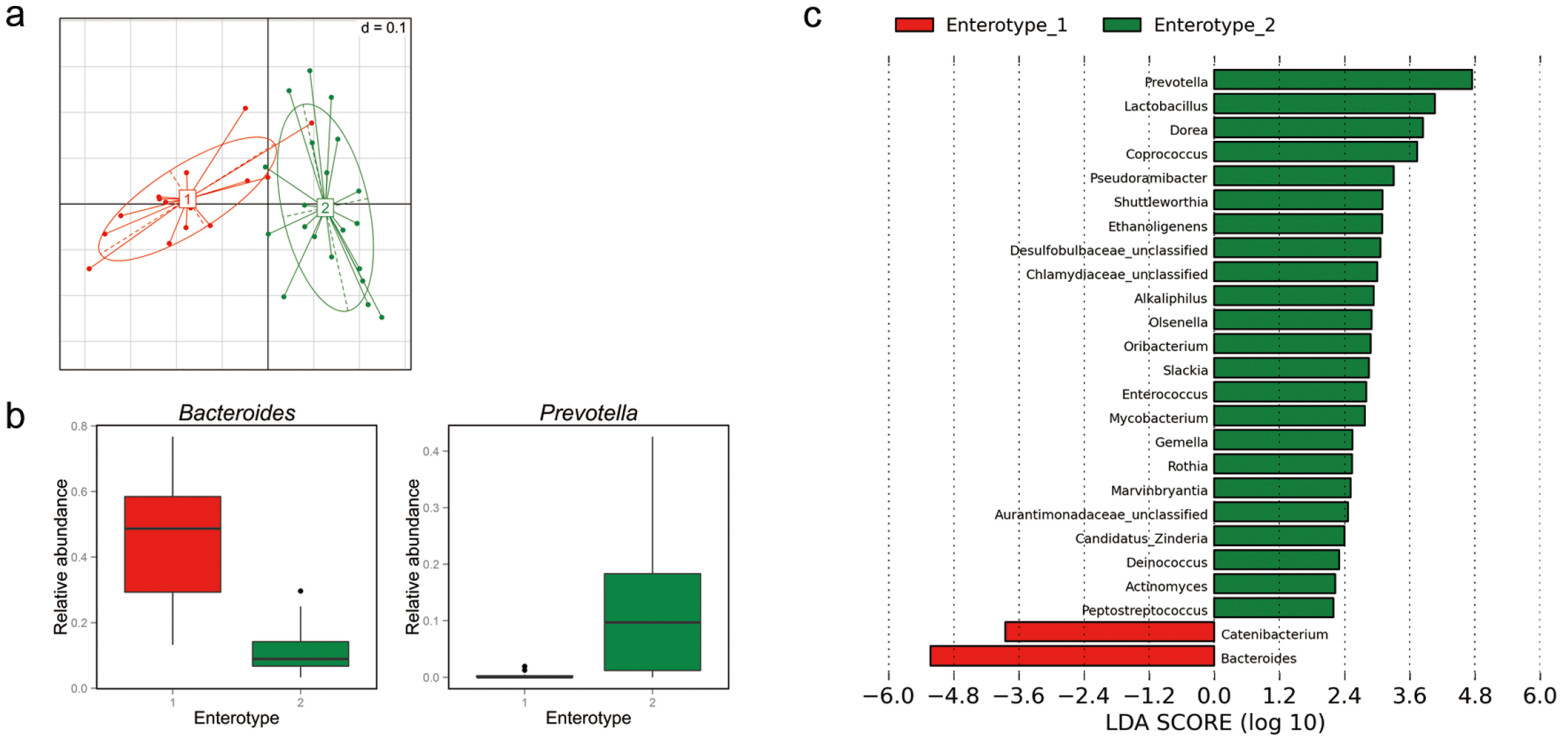
Identification of enterotypes in a Korean population. (a) The first two principal coordinates of the Jensen–Shannon distances of the genus-level relative abundance profiles. Samples are colored by enterotype as identified by the partitioning around medoids (PAM) clustering algorithm. Red is enterotype 1 and green is enterotype 2. (b) Relative abundances of *Bacteroides* and *Prevotella* in each enterotype. Boxes represent the interquartile range (IQR) between the first and third quartiles, with a line at the median. (c) Histogram of the linear discriminant analysis (LDA) score for differentially abundant genera between the enterotypes. Negative (red bars) and positive (green bars) LDA scores represent genera overrepresented in enterotype 1 and enterotype 2, respectively. Features with LDA scores > 2 are presented.

Fifteen (41.7%) of the 36 samples were assigned to enterotype 1, and the rest (58.3%) were assigned to enterotype 2 (Figure 1a and Supplementary Table S1). To identify the genera with different abundance values between the two enterotypes, LEfSe (linear discriminant analysis [LDA] effect size) analysis was performed under the condition α = 0.01, with an LDA score of at least 2. In agreement with previous studies^11^,^14^, enterotype 1 and 2 were enriched in *Bacteroides* and *Prevotella*, respectively (Figure 1b). We also observed that *Catenibacterium* was overrepresented in enterotype 1, whereas 23 genera, including *Lactobacillus*, *Dorea*, and *Coprococcus*, were more abundant in enterotype 2 (Figure 1c). The third enterotype with high abundances of *Ruminococcus*, suggested in the previous study by Arumugam *et al.*^10^, was not observed in this study.

**Figure 2:**
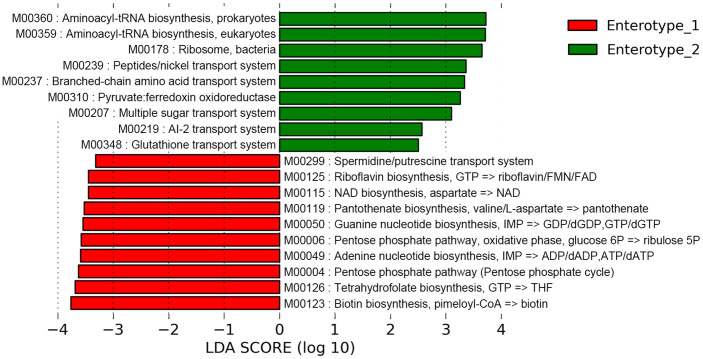
Functional differences between enterotypes. Histogram of the linear discriminant analysis (LDA) scores for differentially abundant KEGG modules between the enterotypes. Negative (red bars) and positive (green bars) LDA scores represent KEGG modules overrepresented in enterotype 1 and enterotype 2, respectively. Features with LDA scores > 2 are presented.

To investigate the functional potential of each enterotype, we applied LEfSe to the relative abundance profiles of KEGG modules. The functional modules involved in the biosynthesis of vitamins, such as riboflavin (vitamin B_2_), pantothenate (vitamin B_5_), biotin (vitamin B_7_), and tetrahydrofolate (vitamin B_9_ derivative), were highly abundant in enterotype 1. Additionally, modules for pentose phosphate pathways and nucleotide (guanine and adenine) biosynthesis were also enriched in enterotype 1 (Figure 2). This observation is in agreement with a recent study showing that the *Prevotella*-dominated (relatively *Bacteroides*-reduced) gut microbiota of patients with untreated rheumatoid arthritis had a reduced abundance of vitamin metabolism and pentose phosphate pathway modules compared to healthy individuals^21^. Thus, *Prevotella* are likely to be key microbes leading to differences in the abundances of these functional modules between the enterotypes. Indeed, the enzymes of the pentose phosphate pathway do not exist in *Prevotella*^22^. In line with our observation of the enrichment of various vitamin biosynthesis modules in enterotype 1, *Bacteroides* living in the gut are known to have a high abundance of genes in the KEGG pathway for vitamin and cofactor metabolism^23^. We also found that enterotype 2 was associated with an increased abundance of transport systems for glutathione, multiple sugars, and branched chain amino acids, which might reflect the high efficiency in the uptake of nutrients from the extracellular environment. At the phylum level, *Firmicutes* have relatively more transport systems than *Bacteroidetes*^5^. Several genera enriched in enterotype 2, including *Lactobacillus*, *Dorea*, and *Coprococcus*, belong to the *Firmicutes*, which may contribute to the enrichment of transport system modules in enterotype 2.

### Genetic and temporal influences on enterotypes

Our subjects consisted of MZ twin pairs, some of whom had longitudinal samples collected after an average interval of 2 years. To determine the genetic and temporal influences on enterotypes, we subsequently calculated the percentage of paired fecal samples from MZ twin pairs that were in the same enterotype at each of the two time points, and the percentage of individuals that belonged to the same enterotype at two different time points.

In terms of the concordance rate between the MZ co-twins, 13 (72.2%) of the 18 paired fecal samples from 10 MZ twin pairs (2 MZ pairs at one time point and 8 MZ twin pairs at two time points) belonged to the same enterotype. Five and eight paired fecal samples from 9 MZ twin pairs belonged to enterotype 1 and enterotype 2, respectively. Among the 18 paired fecal samples, 5 paired fecal samples from 4 MZ twin pairs (3 MZ twin pairs at one time point and 1 twin pair at two time points) were discordant for enterotype, meaning that one sibling of each twin pair was placed in enterotype 1 and the other sibling was placed in enterotype 2 (Table 1). This discordance may be partially caused by the fact that all of our subjects were adults (aged 30–48 years at the first time point), and thus the diets and environments of the twin pairs might not have been shared for a long time. Nevertheless, our data showing that most co-twins had the same enterotype indicate that the enterotype might be affected by the host genetics to some extent.

**Figure 3:**
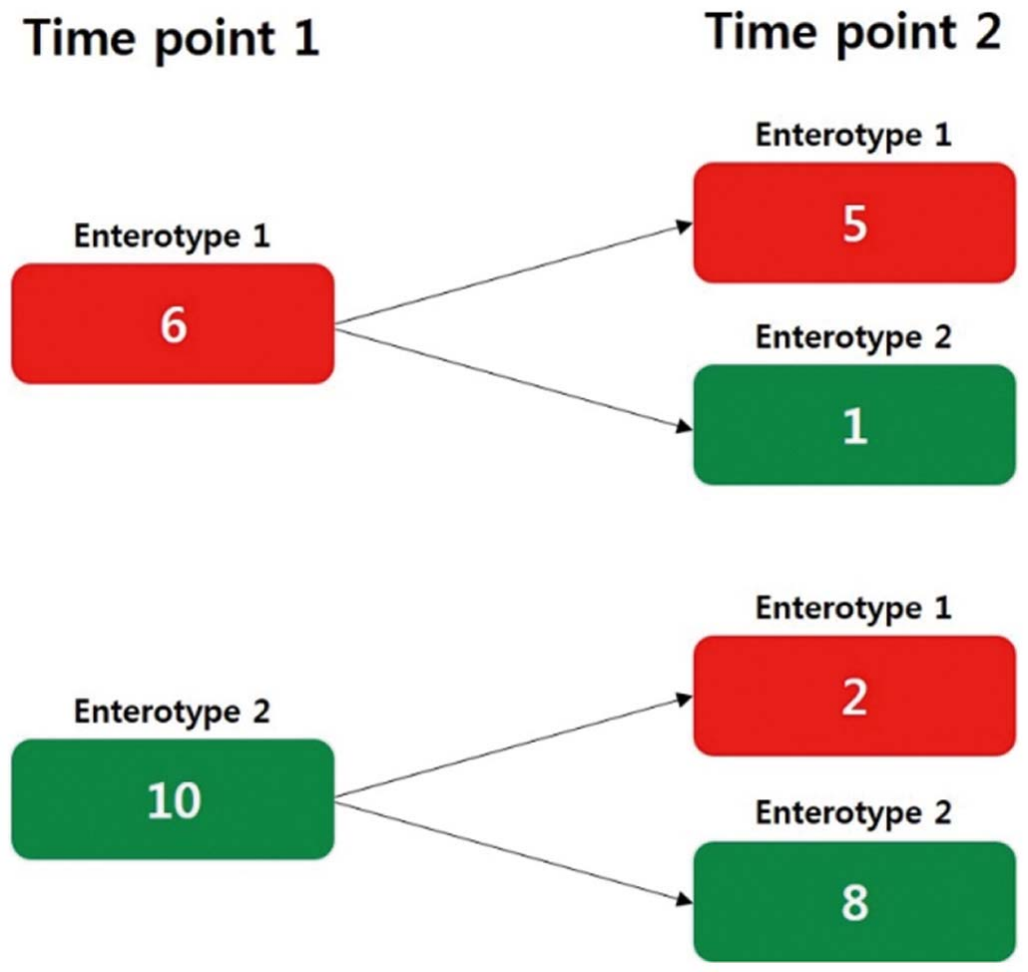
Persistency of enterotypes over time. The numbers of samples assigned to each enterotype at two different time points over the 2-year period are presented.

In terms of the persistency of the enterotype of the same individual after an average of 2 years, 13 (81.3%) of the 16 individuals were assigned to the same enterotype, whereas only 3 (18.7%) of the 16 individuals showed enterotype changes over the 2-year period (Figure 3). This result indicates that the enterotype of each individual is generally stable over time. Previous studies on the stability of enterotypes have suggested that neither a 10-day nor 6-month dietary change was sufficient to lead to enterotype switching^11^,^24^. A recent study with very limited data reported the stability of enterotypes over a long-term period^25^: the enterotypes of all five participating subjects appeared to have changed over a period of 8 or 12 years. To better understand the factors causing the change of an individual's enterotype, larger longitudinal studies with demographic, nutritional, and behavioral data are needed.

To confirm our observation, we additionally compared the JS distances between individuals over time, MZ twin pairs (at the same time point and at two different time points), and unrelated individuals (Supplementary Fig. S2). The results indicated that the JS distance between communities from co-twins at the same time point was shorter than the distance between communities from unrelated individuals, bordering on statistical significance (P = 0.0765). We also observed that the same individual had significantly more similar gut microbial communities over about a 2-year interval compared to those from unrelated individuals (P = 0.0015), which is consistent with previous studies indicating that each individual has a generally stable overall gut microbial community composition over time^5^,^25^,^26^.

### Functional clusters based on KEGG pathway profiles

We further investigated the characteristics of functional clusters based on KEGG pathway abundances. The optimal number of clusters was two, when the JS distance metric was applied (Supplementary Fig. S3). All samples that were classified as enterotype 2 were grouped into functional cluster 2. However, among the 15 fecal samples classified as enterotype 1, only eight were assigned to functional cluster 1, which was comprised of only enterotype 1 samples. The other seven enterotype 1 samples were assigned to functional cluster 2 along with all of the enterotype 2 samples (Supplementary Table S1 and Table 2). These seven samples had relatively low levels of *Bacteroides* and higher levels of genera that were overrepresented in enterotype 2 compared to the rest of the enterotype 1 samples, with an alpha value of 0.05 based on LEfSe (data not shown). This finding suggests that some samples belonging to enterotype 1 appeared to be functionally closer to enterotype 2 than to enterotype 1.

When we performed LEfSe analysis to identify differentially abundant KEGG modules between two functional clusters, we observed that all of the KEGG modules overrepresented in enterotype 1 were also enriched in functional cluster 1 (Supplementary Fig. S4). In functional cluster 2, several additional KEGG modules were enriched, such as threonine and lysine biosynthesis, the maltose/maltodextrin and putative sugar transport systems, and the cobalt transport system, which were not enriched in enterotype 2 based on genus-level abundances. These modules seem to be correlated with the functional properties of gut microbiota from the seven samples, mentioned above in this section. Overall, our results show that the enterotypes based on genus-level abundances do not necessarily correspond with the functional clusters based on metabolic pathway profiles.

### The associations of enterotypes with clinical biomarkers

**Figure 4:**
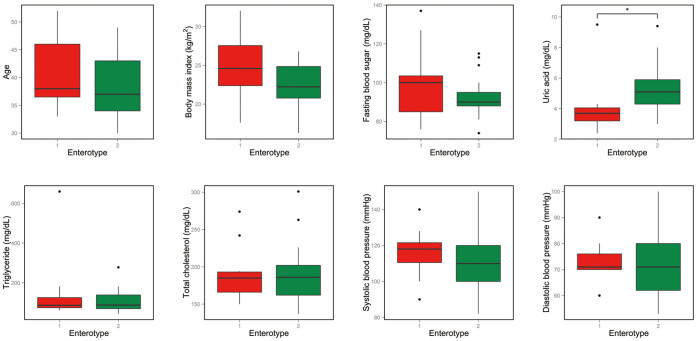
Association of enterotypes with host properties. The Wilcoxon rank-sum test was used to assess the association of enterotypes with age, body mass index, and clinical biomarkers (*adj. P < 0.05). Boxes represent the interquartile range (IQR) between the first and third quartiles, with a line at the median.

Using the Wilcoxon rank-sum test, we investigated the association of enterotypes with host properties including anthropometrical/biochemical measures. The enterotypes were not significantly correlated with biomarkers such as age, BMI, blood pressure, fasting blood sugar, total cholesterol, or triglyceride (Figure 4). The only biomarker found to be significantly different between the enterotypes was the serum uric acid level (adj. P = 0.04). Enterotype 2 had significantly higher levels of uric acid than enterotype 1 (Figure 4). Uric acid is the end product of purine metabolism in humans, and its production is in balance with uric acid disposal under the steady-state condition. Approximately two-thirds of uric acid pool is excreted from the kidney into urine, and the remainder is excreted into gut where uric acid is broken down into allantoin by gut bacteria (intestinal uricolysis)^27^. We found that 5-hydroxyisourate hydrolase (K07127), which is involved in the conversion of uric acid to allantoin, was enriched in enterotype 1 (data not shown). Thus, differences in the functional capacity to metabolize uric acid between the enterotypes may contribute to differences in serum uric acid levels between the enterotypes. Further studies combining metagenomic, metatranscriptomic, and metabolomic approaches are needed to determine which enterotype-associated gut microbes affect serum uric acid levels, and how.

### The associations of enterotypes with long-term diets

**Figure 5:**
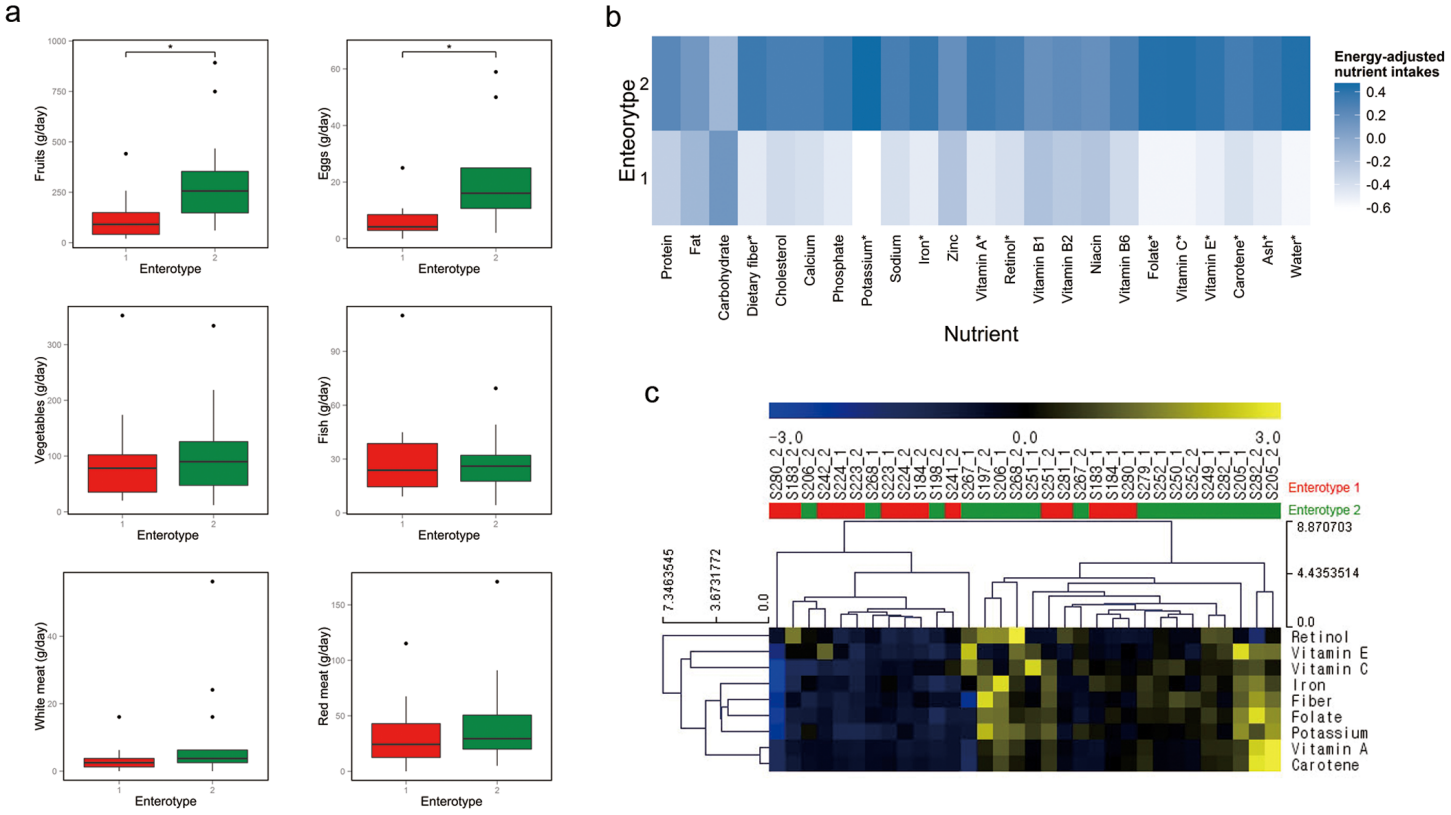
Association of enterotypes with long-term dietary intakes. (a)–(b), The Wilcoxon rank-sum test was used to assess the association of (a) intakes of food items and (b) energy-adjusted nutrient intakes with the enterotypes (*adj. P < 0.05). In (a), boxes represent the interquartile range (IQR) between the first and third quartiles, with a line at the median. In (b), colors represent the mean values of the standardized residuals obtained from the regression of a specific nutrient on energy. Darker blue corresponds to higher intake of the nutrient. (c) Heat map of hierarchical clustering for only the nutrient variables shown to be significantly associated with the enterotypes in this study. Clustering was performed using Euclidean distance and an average linkage method. Red indicates enterotype 1 and green indicates enterotype 2.

We next investigated associations between enterotypes and long-term diets using the Wilcoxon rank-sum test. Enterotypes were previously reported to be associated with long-term dietary patterns: the “*Bacteroides*” enterotype was correlated with a diet enriched in protein and animal fat, while the “*Prevotella*” enterotype was associated with a carbohydrate-enriched diet^11^. In the present study, enterotype 2 was associated with diets higher in fruit and egg food items compared to enterotype 1 (Figure 5a; adj. P = 0.03). For energy-adjusted nutrient intakes, enterotype 2 had significantly higher levels of dietary fiber, minerals (potassium and iron), and vitamins (vitamin A, vitamin C, vitamin E, folate, carotene, and retinol) than enterotype 1 (Figure 5b). No significant associations were noted between enterotypes and macronutrients such as protein, fat, and carbohydrate (Figure 5b).

We next examined the association of overall long-term dietary patterns with the enterotypes. Hierarchical clustering was performed with 23 energy-adjusted nutrient values using EU distance and an average linkage method, which revealed that samples could be clustered into two groups (Supplementary Fig. S5). However, no statistically significant association between two clustered groups using 23 energy-adjusted nutrient values and enterotypes was observed based on Fisher's exact test. On the other hand, when we performed hierarchical clustering using only 9 energy-adjusted nutrient values related to the enterotypes (Figure 5c), we could observe not only the clustering of the samples into two groups, but also the significant association between these groups and the enterotypes (Fisher's exact test, P = 0.03). Therefore, we speculate that the major determinants of the enterotypes are the long-term intakes of dietary fiber and micronutrients, rather than protein, fat, or carbohydrate.

Our data show that enterotype 2 is overrepresented not only by *Prevotella* but also by several bacteria, such as *Lactobacillus* and *Coprococcus*, which have the ability to ferment dietary fiber^28^,^29^. Indeed, De Filippo *et al.* found that the gut microbiome of rural African children was dominated by saccharolytic bacteria, including *Prevotella* and *Xylanibacter*, compared to that of European children^7^. These differences were attributed to the high dietary fiber intake of the rural children. Even though our study subjects were composed of members of a single ethnic group, we were able to observe the association of dietary fiber intake with enterotypes.

The types of vitamins whose intake levels were found to be associated with the enterotypes were vitamin A, including retinol and carotene, vitamin C, and vitamin E (Figure 5b). All of these vitamins have antioxidant properties, and influence immune function by strengthening epithelial barriers and cellular immune responses^30^. Moreover, a study with vitamin A-deficient mice revealed that vitamin A can modulate the composition of gut microbiota, and in turn affect the differentiation of pro-inflammatory Th17 cells^31^. Thus, our data suggest that dietary vitamin intakes may partially contribute to particular configurations of the gut microbiota, and therefore, host immune phenotypes may differ according to the host's enterotype.

We also observed that in enterotype 1, folate intake was lower (Figure 5b) and the KEGG module related to folate biosynthesis was significantly increased (Figure 2). Folate is a water-soluble B vitamin produced by both gut microbes and plants, and is involved in various cellular processes including the synthesis of nucleotides and certain amino acids. Therefore, it is likely that enterotype 1 has adapted to compensate for a dietary shortage of this essential nutrient, in agreement with Yatsunenko *et al*.'s observation that the genes related to the riboflavin biosynthesis are more abundant in the gut microbiome of the Malawian and Amerindian infants whose vitamin availability is lower than the U.S. infants^6^.

With regard to minerals, higher dietary iron intake was found to be associated with enterotype 2 (Figure 5b). Iron is an essential micronutrient for most of the gut microbiota, and the ability to acquire iron differs among bacterial strains. Several studies have reported that iron supplementation leads to compositional changes in the gut microbiota in humans and in animal models^32^,^33^,^34^. Thus, the gut microbial compositions forming the different enterotypes could be influenced by dietary iron intake.

Finally, for enterotype-discordant twin pairs, we performed the Wilcoxon signed-rank test to identify significantly different host characteristics including anthropometrical, biochemical, and dietary features. No significant difference was found at a false discovery rate of 5%. Similarly, no significant differences were observed in host characteristics between functional clusters based on KEGG pathway profiles.

## Conclusions

In this study, we presented that the gut microbiota of a Korean MZ twin population can be categorized into two enterotypes, each enriched in either *Bacteroides* or *Prevotella*. Our longitudinal samples of MZ twins showed that most co-twins shared their enterotypes, and that the enterotype of an individual remained mostly unchanged over a long-term period. The serum uric acid level and the long-term intakes of nutrients such as dietary fiber, vitamins, and minerals were found to be associated with the subjects' enterotypes.

## Methods

### Ethics statement

All experiments were performed in accordance with relevant guidelines and regulations approved by the institutional review board of Samsung Medical Center, Busan Paik Hospital, and Seoul National University (IRB No. 144-2011-07-11). Written informed consent was obtained from all participants.

### Sample collection

Thirty-six fecal samples were obtained from 20 MZ twins (10 MZ twin pairs) enrolled in the Healthy Twin Study in Korea^35^. Among them, 16 MZ twins (8 MZ twin pairs) provided fecal samples at two time points with an average interval of 2 years, and 4 MZ twins (2 MZ twin pairs) provided samples at only one time point. Therefore, 36 fecal samples were collected and subsequently analyzed. The fecal samples were produced at home and immediately placed in freezer. Frozen fecal samples were brought to clinics and stored at −80°C prior to subsequent analysis.

### Metadata collection

Anthropometrical measurements and biochemical tests were conducted for each subject at every visit. Weight and height were measured using the InBody 3.0 (Biospace Inc., Seoul, Korea) body composition analyzer, and BMI was calculated as the weight (kg) divided by the square of the height (m^2^). Blood pressure was measured with a standard mercury sphygmomanometer. Blood samples were drawn from an antecubital vein after an overnight fast of at least 8 h and sent to one central laboratory for blood tests within 24 h. Measurements of glucose, total cholesterol, triglyceride, high-density lipoprotein, and low-density lipoprotein were assessed by enzymatic methods. Insulin levels were measured by a radioimmunoassay method. The serum uric acid level was assayed by a uricase enzymatic colorimetric method, and high-sensitive C-reactive protein was measured by latex agglutination.

Participants also filled out questionnaires covering lifestyle, medication, and disease history. Long-term dietary information was obtained using a 106-item food frequency questionnaire for Koreans^36^. For each food item, the intake in grams per day was estimated based on the consumption frequency (nine categories: never or seldom, once a month, two to three times a month, once or twice a week, three to four times a week, five to six times a week, once a day, twice a day, or three times or more every day) and the portion size (three categories: small, medium, or large). Average daily intakes of energy and 23 nutrients including protein, fat, carbohydrates, fiber, vitamins, and minerals were calculated using a computer program (Korean Genomic Epidemiological Cohort Study Information System, version 1.0), but this information was missing for four of the 36 samples. For the analyses, we used the average daily intake for each of the 30 food groups that were created by combining similar food items into a single group. We also used energy-adjusted nutrient intakes that were calculated as the standardized residuals from the regression of a specific nutrient on energy.

### Metagenomic shotgun sequencing and analysis

DNA extraction from each fecal sample was conducted using the MoBio Power Soil DNA Isolation Kit (MoBio, Solana Beach, CA, USA). All samples were sequenced by 101-bp paired-end sequencing on a HiSeq platform (Illumina, San Diego, CA, USA) according to the manufacturer's instructions. The raw reads were filtered to remove low-quality reads and human sequence contamination. To estimate the taxonomic composition of each sample, the filtered reads were mapped to a set of clade-specific marker genes using MetaPhlAn^37^. For metabolic reconstruction, high-quality reads were mapped to the protein-coding sequences from the KEGG Orthology using USEARCH, and best hits were further passed through HUMAnN^38^.

### Enterotyping and functional clustering

For enterotyping and functional clustering of the gut microbiota, we calculated JS, BC, and EU distances for the genus-level relative abundance profiles and for the KEGG pathway relative abundance profiles. To cluster the samples based on these distance metrics, we used the PAM method in the R package ‘cluster.’ The optimal number of clusters was chosen based on CH and SI values that were calculated using the R package ‘clusterSim.’ The samples were plotted by principal coordinate analysis using the R package ‘ade4.’

### Statistical analysis

To determine taxonomic and metabolic features that were differentially abundant either between enterotypes or between functional clusters, LEfSe was applied^39^. The concordance rate for the enterotype in MZ twins was calculated as the percentage of paired fecal samples from MZ twin pairs that were assigned to the same enterotype at each time point. Similarly, the persistency rate of the enterotype over time was calculated as the percentage of individuals who were assigned to the same enterotype at two different time points. Two-sample permutation tests were conducted on JS distances of the gut microbiota based on the genus-level relative abundances for the four types of comparisons for all samples obtained from each individual: an individual at different time points, twin pairs at the same time point, twin pairs at different time points, and unrelated individuals. Differences in anthropometrical/biochemical biomarker measures and long-term diets (both dietary patterns and nutritional factors) between enterotypes were analyzed using the nonparametric Wilcoxon rank-sum test. To identify host properties that demonstrated significantly different abundances between co-twins discordant for enterotype, we applied the Wilcoxon signed-rank test. Heat maps of the energy-adjusted nutrient intakes across samples were generated using the Multi-Experiment Viewer software (version 4.8.1). Hierarchical clustering of samples was performed with the energy-adjusted nutrient intake data using EU distance and an average linkage method. Fisher's exact test was used to determine associations between enterotypes and groups clustered based on long-term patterns of nutrient intakes. P-values were adjusted for multiple testing with the Benjamini–Hochberg method.

## Author Contributions

M.L., M.R. and G.K. conceived and designed the experiments. M.L. performed the experiments, analyzed the data, and wrote the paper. Y.S., K.L. and J.S. performed sample collection. All authors reviewed the manuscript.

## Supplementary Material

Supplementary Information Supplementary information

## Figures and Tables

**Table 1:** Enterotype assignment of the paired fecal samples from the MZ twin pairs at each of the two time points

	Concordant paired fecal samples		Discordant paired fecal samples		Total	
Enterotype	N	%	N	%	N	%
**1**	5	27.8	-	-	5	27.8
**2**	8	44.4	-	-	8	44.4
**1/2**	-	-	5	27.8	5	27.8
**Total**	13	72.2	5	27.8	18	100.0

**Table 2:** Distribution of samples based on enterotypes and functional clusters

	Community composition					
	Enterotype 1		Enterotype 2		Total	
Metabolic pathway	N	%	N	%	N	%
**Functional cluster 1**	8	22.2	0	0.0	8	22.2
**Functional cluster 2**	7	19.4	21	58.3	28	77.8
**Total**	15	41.7	21	58.3	36	100.0

## Notes

**Accession codes:** The sequences from this study were deposited in the European Nucleotide Archive under the study accession number ERP002391.
