# Supplementary material for: Stability of Gut Enterotypes in Korean Monozygotic Twins and Their Association with Biomarkers and Diet
Source: Sci Rep. 2014 Dec 8;4:7348. doi: 10.1038/srep07348 (PMC4258686; doi:10.1038/srep07348)
Supplement: Supplementary Information [file srep07348-s1.pdf]

Stability of Gut Enterotypes in Korean Monozygotic Twins and Their Association with Biomarkers and Diet

Mi Young Lim, Mina Rho, Yun-Mi Song, Kayoung Lee, Joohon Sung, GwangPyo Ko

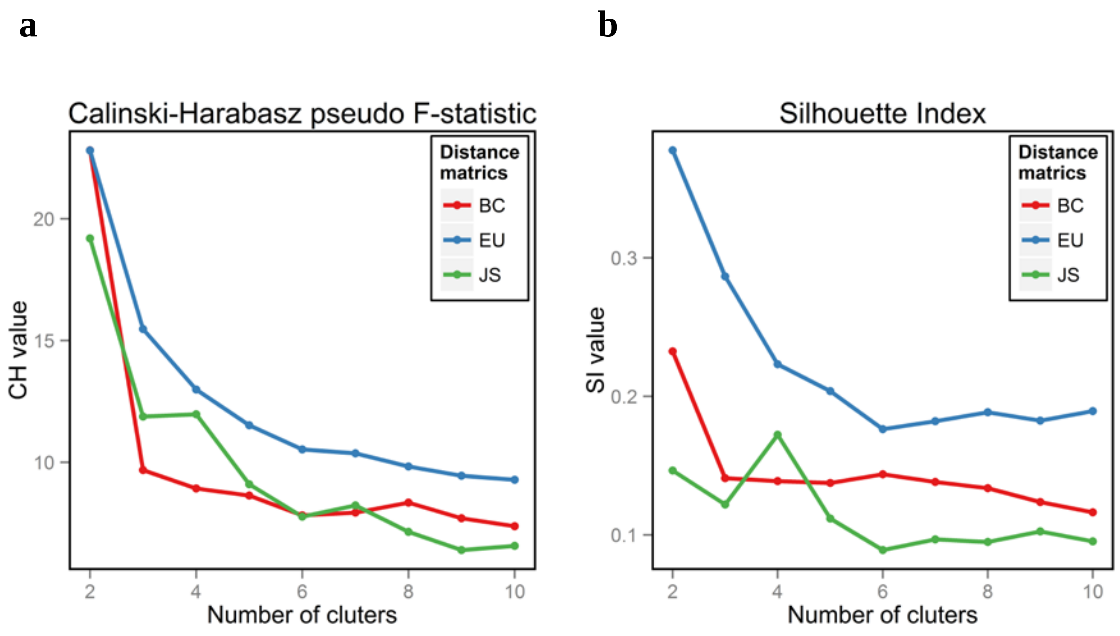

Supplementary Fig. S1 Estimation of the optimal number of enterotypes. (a) The Calinski–Harabasz pseudo *F*-statistic and (b) Rousseeuw's Silhouette internal cluster quality index for three distance metrics (Jensen–Shannon [JS], Bray–Curtis [BC], Euclidean [EU] distances) of the genus-level relative abundance profiles.

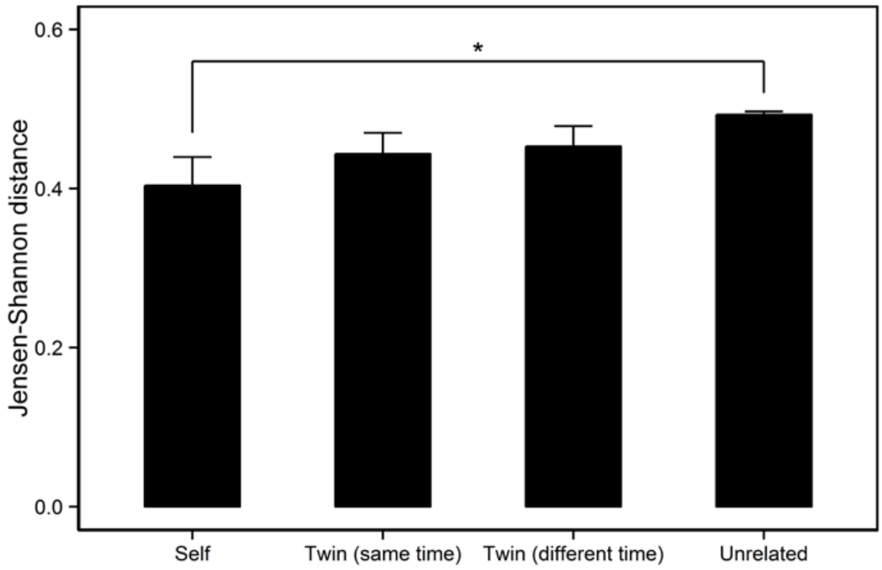

Supplementary Fig. S2 Jensen–Shannon distances of the genus-level relative abundance profiles between individuals over time (self), twin pairs (at the same time point and at two different time points), and unrelated individuals (two sample permutation test; *$P < 0.05$; mean ± s.e.m.).

a

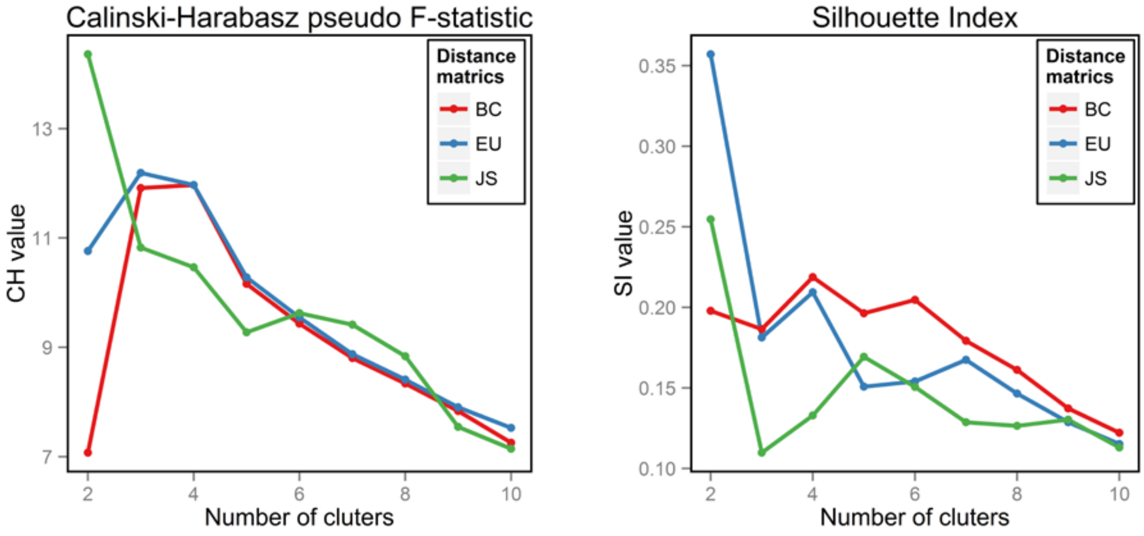

b

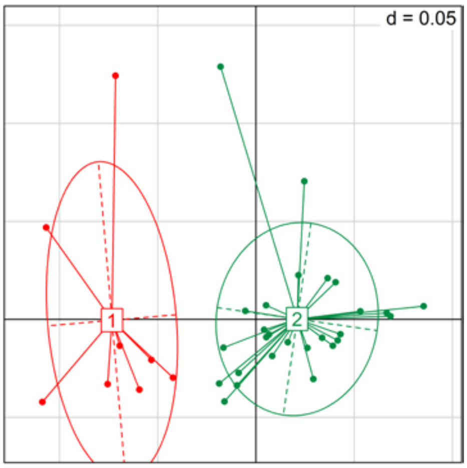

Supplementary Fig. S3 Identification of two functional clusters based on KEGG pathway profiles. (a) Estimation of the optimal number of functional clusters calculated from the Calinski–Harabasz pseudo *F*-statistic and Rousseeuw's Silhouette internal cluster quality index for three distance metrics (Jensen–Shannon [JS], Bray–Curtis [BC], Euclidean [EU] distances) of the KEGG pathway relative abundance profiles, and (b) the first two principal

coordinates of the Jensen–Shannon distances of the KEGG pathway profiles. Samples are colored by functional cluster as identified by the partitioning around medoids (PAM) clustering algorithm. Red is functional cluster 1 and green is functional cluster 2.

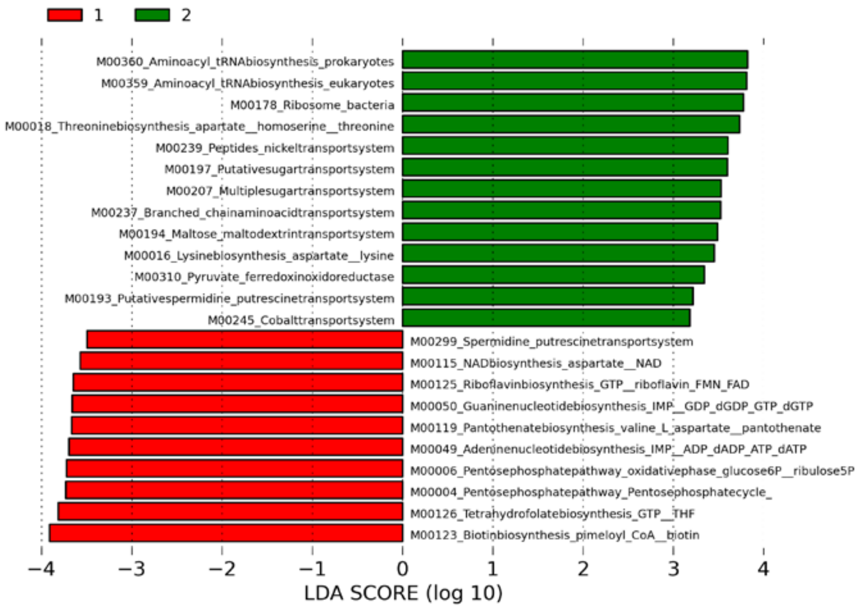

Supplementary Fig. S4 Functional differences between two functional clusters based on KEGG pathway profiles. Histogram of the linear discriminant analysis (LDA) scores for differentially abundant KEGG modules between the functional clusters. Negative (red bars) and positive (green bars) LDA scores represent KEGG modules overrepresented in functional cluster 1 and functional cluster 2, respectively. Features with LDA scores >2 are presented.

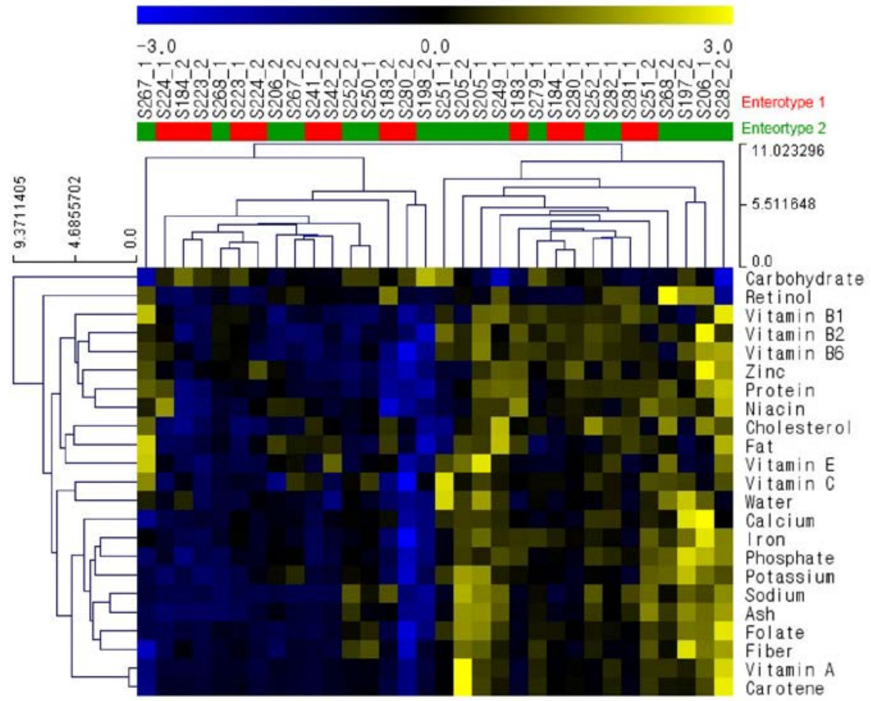

Supplementary Fig. S5 Heat map of hierarchical clustering for 23 energy-adjusted nutrient values. Clustering was performed using Euclidean distance and an average linkage method. Red indicates enterotype 1 and green indicates enterotype 2.

Supplementary Table S1 Sample assignment to the enterotypes for three distance metrics (Jensen–Shannon [JS], Bray–Curtis [BC], Euclidean [EU] distances) and to the functional clusters based on JS distance.

SampleID	Twin	Enterotype			Functional cluster
		JS	EU	BC	JS
S183_1	T92	1	1	1	2
S183_2	T92	1	1	1	1
S184_1	T92	1	1	1	1
S184_2	T92	1	1	1	1
S197_2	T99	2	2	2	2
S198_2	T99	2	2	2	2
S205_1	T103	2	2	2	2
S205_2	T103	2	2	2	2
S206_1	T103	2	2	2	2
S206_2	T103	2	2	2	2
S223_1	T112	1	1	1	2
S223_2	T112	1	1	1	2
S224_1	T112	1	1	2	2
S224_2	T112	1	1	1	1
S241_2	T121	1	1	1	1
S242_2	T121	1	1	1	1
S249_1	T125	2	2	2	2
S249_2	T125	1	1	2	2
S250_1	T125	2	2	2	2
S250_2	T125	2	2	2	2
S251_1	T126	2	2	2	2
S251_2	T126	1	1	2	2
S252_1	T126	2	2	2	2
S252_2	T126	2	2	2	2
S267_1	T134	2	2	2	2
S267_2	T134	2	2	2	2
S268_1	T134	2	2	2	2
S268_2	T134	2	2	2	2
S279_1	T140	2	2	2	2
S279_2	T140	2	2	2	2
S280_1	T140	1	1	1	2
S280_2	T140	1	1	1	1
S281_1	T141	1	1	1	1
S281_2	T141	2	2	2	2
S282_1	T141	2	2	2	2
S282_2	T141	2	2	2	2
